# Meta-analysis of association between c.963A*>*G single-nucleotide polymorphism on *BMP15* gene and litter size in goats

**DOI:** 10.5194/aab-65-309-2022

**Published:** 2022-08-12

**Authors:** Emel Zergani, Amir Rashidi, Jalal Rostamzadeh, Mohammad Razmkabir, Jens Tetens

**Affiliations:** 1 Department of Animal Science, Faculty of Agriculture, University of Kurdistan, Sanandaj, 66177, Iran; 2 Department of Animal Sciences, Faculty of Agricultural Sciences, University of Göttingen, 37077 Göttingen, Germany

## Abstract

Litter size is an important economic trait in the goat industry. Previous studies on the bone morphogenetic protein 15 (*BMP15*) gene detected some single-nucleotide polymorphisms (SNPs) such as c.963A > G that were associated with an increase in ovulation rate and litter size. The aim of this study was to conduct a meta-analysis on the effect of this polymorphism on litter size. We gathered and pooled data from five eligible published studies. To investigate the effect of c.963A > G on litter size, we utilized four different genetic models assuming dominant (*GG* + *GA* vs. *AA*), recessive (*GG* vs. *GA* + *AA*), additive (*GG* vs. *AA*) and co-dominant (*GG* + *AA* vs. *GA*) model of inheritance. Data were analyzed under random-effects models based on the $I^{2}$ value. Furthermore, sensitivity analysis was carried out to validate the stability of results. The results showed that the c.963A > G polymorphism is associated with litter size when applying a dominant model (standardized mean difference (SMD) is 0.815, 95 % CI [0.170, 1.461], P value = 0.013) and also with an additive model (SMD = 0.755, 95 % CI [0.111, 1.400], P value = 0.022). However, the effect of c.963A > G polymorphism was not significant under recessive (SMD = 0.186, 95 % CI [ - 0.195, 4.259], P value = 0.339) and co-dominant (SMD = - 0.119, 95 % CI [ - 0.525, 0.288], P value = 0.568) models. Sensitivity analysis demonstrated that dropping studies with wide confidence intervals affects overall results under the assumption of an additive model. The meta-analysis results revealed that the *AA* genotype could be positively connected with litter size in goats.

## 1 Introduction

Goats are spread all around the world, especially in harsh and marginal regions. They play an important economic role in developing countries (Araújo et al., 2010). The goat population is increasing in developing countries due to their different food consumption patterns and lower water requirements in comparison with other livestock species such as cattle and sheep (Moghadaszadeh et al., 2015). Goats are raised for meat, milk and hair, particularly mohair or cashmere production, and it is clear that highly productive goats can improve the quality and increase the quantity of the mentioned products (Jalbani et al., 2017).

In recent years, the improvement of reproductive traits, such as litter size (LS), has become one of the great interests of breeders and local farmers, and consequently, research efforts have been made to unravel these traits' genetic basis (Eghbalsaied et al., 2009). Although litter size is a complex trait influenced by numerous genes and environmental factors, some major genes have been identified to influence litter size (Lai et al., 2016). Among them is the bone morphogenetic protein 15 (*BMP15*) that regulates the proliferation and differentiation of granulosa cells by stimulating their mitosis, stopping the expression of the *FSH* gene receptor and expressing the stimulation of the ligand. The protein plays a major role in female fertility in mammals (Juengel et al., 2002). This gene is located on the X chromosome and comprises two exons with 1185 base pairs in total (Ahlawat et al., 2016). Studies performed in different prolific goat breeds have indicated that some *BMP15* variants increase ovulation rates and subsequently litter size (Pramod et al., 2013). A mutation study of this gene suggested that even exchanging an amino acid that does not cause a large alteration in the product sequence can lead to a large impact on the activity of the product, followed by the ovulation rate (Hanrahan et al., 2004).

For the variant c.963A > G in exon 2 of the *BMP15* gene, ambiguous effects have been reported. Some researchers observed a significant effect of c.963A > G on litter size in goats (Chu et al., 2007; Feng et al., 2009; Dong and Du, 2010; Feng et al., 2014; Moghadaszadeh et al., 2015), whereas others did not find any association between this single-nucleotide polymorphism (SNP) and litter size (Dong and Du, 2010). Because of small sample sizes, some of the mentioned studies reported low statistical power to validate negative or positive effects of c.963A > G variants on litter size. Therefore, the power of a meta-analysis can overcome the low-sample-size issue and increase the validity of the effect of c.963A > G polymorphism on litter size in goats.

These studies on fertility traits in livestock, especially on the *BMP15* gene in goats, encouraged us to conduct a meta-analysis on the mutation c.963A > G, which had the greatest impact on fertility traits, and collect all reported studies on this gene and mutation.

Meta-analysis is a quantitative and formal study design used to assess the previous findings of researchers about specific questions to obtain a more validated conclusion about that type of research. Outcomes from a meta-analysis can provide a more precise estimate of the effect of treatments or other factors on a trait than any single study because of pooled results included in the analysis (Lean et al., 2009). Some meta-analyses have been conducted on litter size in goats (Mahmoudi et al., 2019) and milk-related traits in cattle (Mahmoudi et al., 2020) and small ruminants (Razmkabir et al., 2021). To the best of our knowledge, no meta-analysis has been conducted on association of detected SNPs in the *BMP15* gene with reproductive traits in goats. Therefore, the objective of this study was to conduct a meta-analysis by pooling all results reported in different studies in scientific journals in order to investigate the effect of c.963A > G polymorphism on litter size in goats.

## 2 Material and methods

### 2.1 Search strategy for identification of relevant studies

The preferred reporting items for systematic reviews and meta-analyses (PRISMA) checklist criteria were used to identify eligible studies for this meta-analysis. Two investigators (Emel Zergani and Jalal Rostamzadeh) independently searched databases including Springer, ScienceDirect, Wiley and PubMed to detect studies relevant to our question using combination of search terms as follows: “BMP15”, “SNP”, “polymorphism”, “prolificacy”, “litter size”, “capra hircus” and “goat”. Furthermore, we explored all Chinese and Persian journals and databases to find articles published in different languages. In addition, we scrutinized reference lists of extracted articles to assure that no articles were missed. All articles which were in the form of an abstract or review and also any kind of duplication were removed, and the quality of remaining full-text articles was appraised by two investigators. Finally, the third investigator (Amir Rashidi) resolved all conflicts and disagreements for inclusion and exclusion of studies.

**Table 1:** Characteristics of studies included in this meta-analysis.

First author	Year of publication	Goat breed	Total sample	Genotypes			LSM ± SE			Significant
				*GG*	*GA*	*AA*	*GG*	*GA*	*AA*	
Chu	2007	Jining Grey	100	0	90	10	NE	2.58 ± 0.14	1.45 ± 0.11	Yes
Feng	2009	Jining Grey	135	126	8	1	2.67 ± 0.07	1.96 ± 0.12	1.1 ± 0.03	Yes
Dong	2010	Jining Grey	201	136	34	31	2.71 ± 0.06	2.73 ± 0.11	2.76 ± 0.11	No
Dong	2010	Lubei White	51	17	24	10	2.56 ± 0.09	2.54 ± 0.08	2.23 ± 0.12	Yes
Dong	2010	Yimeng Black	74	8	23	43	1.17 ± 0.08	1.18 ± 0.04	1.06 ± 0.03	Yes
Feng	2014	Jining Grey	211	189	19	3	2.83 ± 0.08	2.18 ± 0.15	1.08 ± 0.06	Yes
Moghadaszadeh	2015	Raini Cashmere	200	16	84	100	0.66 ± 0.28	1.95 ± 0.07	1.66 ± 0.08	Yes

NE: not existent.

**Table 2:** The heterogeneity test results for genetic models.

Genetic model	Heterogeneity analysis			Selected model
	Q	P value	$I^{2}$ (%)	
Dominant (*GG* + *GA* vs. *AA*)	25.272	0.000	84.172	Random
Recessive (*GG* vs. *GA* + *AA*)	27.783	0.000	74.404	Random
Additive (*GG* vs. *AA*)	18.683	0.001	78.590	Random
Co-dominant (*GG* + *AA* vs. *GA*)	31.262	0.000	80.808	Random

**Table 3:** The outcomes of meta-analysis of the association between the c.963A > G polymorphism and the litter size under different genetic models.

Genetic model	No. breeds	SMD	95 % confidence interval		P value
			Lower limit	Upper limit	
Dominant (*GG* + *GA* vs. *AA*)	5	0.815	0.170	1.461	0.013
Recessive (*GG* vs. *GA* + *AA*)	7	0.186	- 0.195	4.259	0.339
Additive (*GG* vs. *AA*)	5	0.755	0.111	1.400	0.022
Co-dominant (*GG* + *AA* vs. *GA*)	7	- 0.119	- 0.525	0.288	0.568

SMD: standardized mean difference.

**Table 4:**
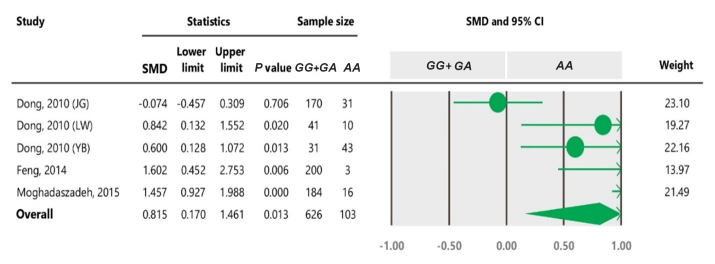
Forest plot of association between c.963A > G polymorphism and the litter size under the dominant model. The size of green circles represents the weight of each study. The horizontal green line shows the confidence interval for each study. The diamond located in the bottom of plot represents the summary result. The name given in “Study” column refers to the first author of the respective study. JG denotes Jining Grey; LW denotes Lubei White; YB denotes Yimeng Black.

### 2.2 Inclusion and exclusion criteria

Studies were eligible if they met the following criteria: (1) report on c.963A > G single-nucleotide polymorphism, (2) provide the sample size for each genotype, (3) investigate association between c.963A > G SNP and litter size, (4) report the least-squares mean (LSM) for each genotype, and (5) report the standard error for each reported LSM of genotypes. The criteria for exclusion studies were as follows: (1) studies which were in the form of an abstract, (2) studies with insufficient data, (3) duplicate articles and (4) review articles.

### 2.3 Data extraction

The data included in our meta-analysis were extracted from selected studies based on designated inclusion and exclusion criteria. The extracted data included the first name of the author, year of publication, goat breed and sample size, LSM, and standard error reported for each genotype.

Considering that the standard deviations are needed to analyze data, we employed the following equation to calculate SD from sample sizes of genotypes and standard errors of the LSM:

$$\mathrm{SD}=\mathrm{SE}\sqrt{N},$$

where SE is the standard error of the mean reported for the genotype and N is the sample size of the genotype. For combined genotypes, pooled LSMs and SDs were computed using the approach described in the *Cochrane Handbook for Systematic Reviews of Interventions* (Higgins and Green, 2011).

### 2.4 Statistical analysis

ReviewManager v5.0 software was used to analyze data collected from different studies employing recessive (*GG* vs. *GA* + *AA*), dominant (*GG* + *GA* vs. *AA*), additive (*GG* vs. *AA*) and co-dominant (*GG* + *AA* vs. *GA*) genetic models.

In the next stage, Cochran's Q test ( P<0.01 considered to be significant) was used to evaluate the pattern of heterogeneity among studies included in this meta-analysis. It is suggested that a non-significant value for the Q test does not necessarily indicate the same population for included studies because of a small sample size for the comparisons and a small number of comparisons contributing to the meta-analysis (Vesterinen et al., 2014). For this reason, the $I^{2}$ statistic with a range from 0 % to 100 % was additionally used to quantify heterogeneity of included studies. Then we fitted a fixed-effects model to analyze data when the heterogeneity was low ( $I^{2}$ < 50 %), and a random-effects model was used when the heterogeneity was high ( $I^{2}$ > 50 %). To detect the stability of overall results, we performed a sensitivity analysis by dropping a single study at a time. Finally, we carried out Egger's linear regression test and produced a funnel plot to detect publication bias among studies.

## 3 Results

### 3.1 Characteristics of included studies

This method of studies contains the primary components of a systematic review and meta-analysis. The identification stage is the first stage, and second is the development of a detailed protocol and its preregistration. Searching two literature databases at least, along with other sources of published studies (using reviews, field experts, own data, non-English literature), is recommended. It is necessary to mention search dates and exact keyword threads.

**Figure 1:**
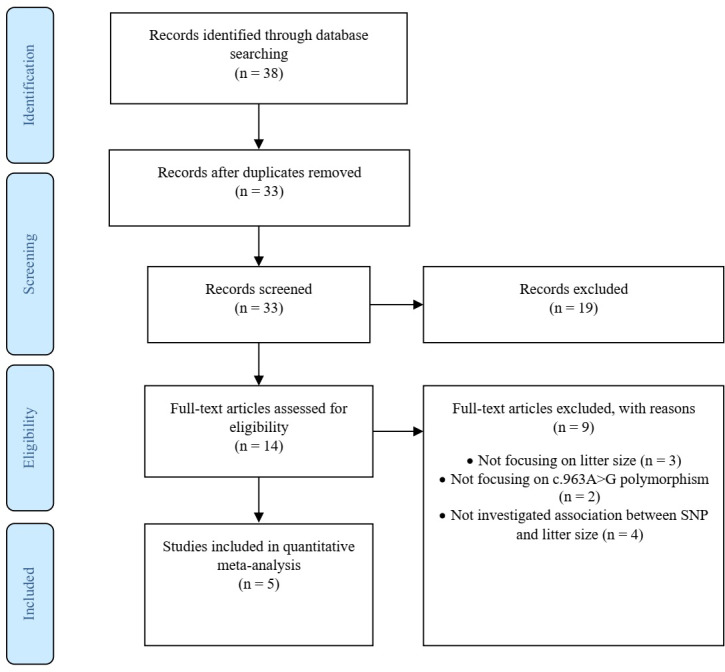
The PRISMA flowchart showing inclusion and exclusion criteria.

The screening and eligibility stage should be based on the inclusion and exclusion criteria studies. Criteria might differ for the initial screening (title, abstract) compared to the full-text screening, but both need to be reported in detail. At least two investigators should study and decide on the selection of eligible articles, with a plan for disagreement resolution and calculating disagreement rates. The list of studies excluded at the full-text screening stage, with reasons for their exclusion, and a full list of studies included in the final dataset, with their basic characteristics, are reported. We recorded the figures and tables as well as reported intermediate calculations, transformations, simplifications and assumptions made during data extraction. These details make identifying mistakes easier and modify reproducibility. Documentation included a summary of the dataset, information on data and study details that authors reported, a short explanation of software used for analyses. Therefore, we created a PRISMA diagram (Fig. 1), which records the starting information from the studies and leads to the final dataset (Nakagawa et al., 2017).

A total of 38 articles were identified through search on databases including PubMed, ScienceDirect, Wiley Online Library, CNKI (Chinese) and Magiran (Iranian).

In addition to the abstracts, a total of five duplicate studies were removed. Then, we screened the remaining publications to exclude irrelevant studies, resulting in deletion of nine articles which did not investigate the SNP and/or trait of interest. Furthermore, in some studies polymorphisms have been reported, but their association with litter size was not evaluated; thus these studies were also rejected. In conclusion, five studies involving 978 goats were selected to be included in our meta-analysis, three of which were written in English, one in Persian and the last one in Chinese. Among the selected studies, two pieces of research were conducted on different breeds of goats; thus each breed was evaluated as a separate study in the meta-analysis. Characteristics of included studies are presented in Table 1.

### 3.2 Evaluation of heterogeneity among studies

Table 2 involves Cochran's Q heterogeneity test and results of the $I^{2}$ statistic for four genetic models. The calculated $I^{2}$ for all genetic models was greater than 50 %. Hence the random-effects model was used to investigate the association between c.963A > G polymorphism and litter size in goats.

### 3.3 Meta-analysis of the relationship between the c.963A > G polymorphism and litter size

The results of meta-analysis of association between the SNP and trait of interest under four genetic models are summarized in Tables 3–7. The estimates did not show any association between the c.963A > G polymorphism and litter size under a recessive (SMD = 0.186, 95 % CI [ - 0.195, 4.259]) or co-dominant (SMD = - 0.119, 95 % CI [ - 0.525, 0.288]) model. However, the obtained results showed a significant ( P<0.05 ) association between the c.963A > G polymorphism and the litter size under dominant (SMD = 0.815, 95 % CI [0.170, 1.461]) and additive (SMD = 0.755, 95 % CI [0.111, 1.400]) genetic models.

### 3.4 Sensitivity analysis and publication bias

**Figure 2:**
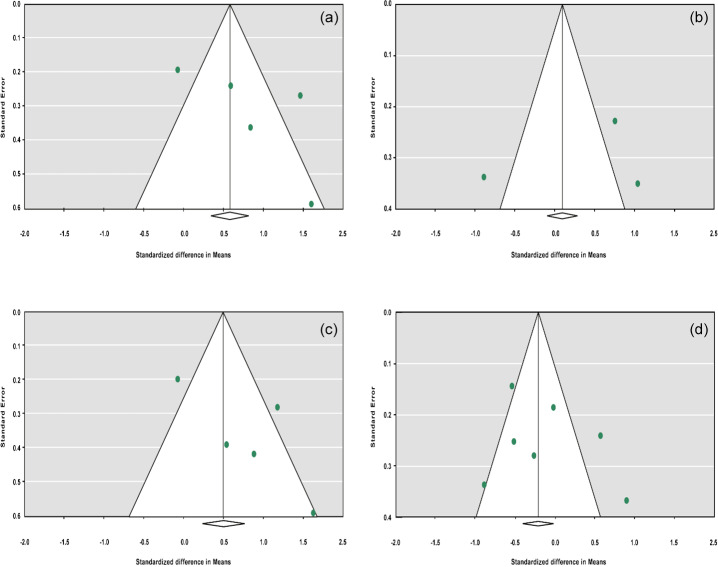
Funnel plots for the publication bias under the dominant model **(a)**, recessive model **(b)**, additive model **(c)** and co-dominant model **(d)**.

**Figure 3:**
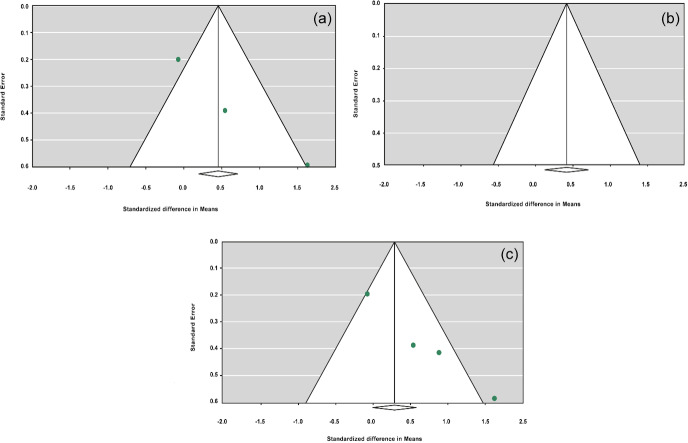
Funnel plots of sensitivity analysis under the additive model, with the Dong and Du (2010) Lubei White breed study removed **(a)**, the Feng et al. (2014) study removed **(b)**, and the Moghadaszadeh et al. (2015) study removed **(c)**.

The sensitivity analysis was performed to investigate the robustness and validity of the meta-analysis using a leave-one-out approach. We did not observe any difference in pooled results of SMDs before and after removing one study in dominant, recessive, additive and co-dominant genetic models. The funnel plots for studies drawn in all genetic models are depicted in Fig. 2. As is observable, the shape of all plots indicates no publication bias under all four employed models. On the contrary, sensitivity analysis showed significant difference in litter size by dropping studies performed by Dong and Du (2010) on the Lubei White breed and by Feng et al. (2014) and Moghadaszadeh et al. (2015) under the additive model (Table 8 and Fig. 3). Furthermore, the results of Egger's regression test obtained for all four comparison models showed no evidence of publication bias at the level of P<0.05 .

## 4 Discussion

It is important to understand the genetic regulation of reproduction traits in livestock (Nicol et al., 2009). The *BMP15* gene is essential for female fertility, so any knowledge of its function allows breeders to improve the ovulation rate and litter size in farm animals. In addition, the study of genes encoding reproductive proteins is also important for capturing information on genetic disorders associated with reproduction traits (Pramod et al., 2013). A reduction in the β error and increase in the accuracy of effect estimation are benefits of meta-analysis; however, the main problem of meta-analysis is the probable heterogeneity among studies, which requires a strict study design.

Association of the litter size with some important genes, especially *BMP15*, in goats has been examined (Wang et al., 2011). *BMP15* is an X chromosomal gene known as the *FecX* gene (fecundity X gene) that is associated with litter size (Lassoued et al., 2017). Given the large effect of *BMP15* mutations on ovulation and litter size, it can be regarded as a major gene for reproduction in farm animals (Nagdy et al., 2018).

**Table 5:**
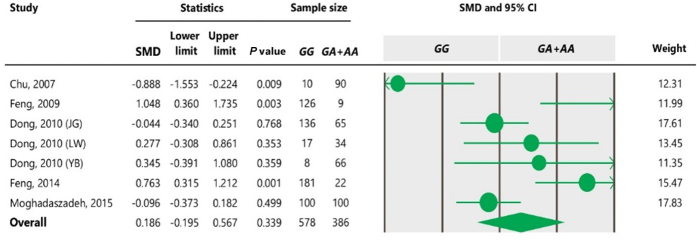
Forest plot of association between c.963A > G polymorphism and the litter size under the recessive model. For additional details refer to the Table 4 caption.

In research on genetic mutations and effects on the ovulation rate in sheep, results have shown that *BMP15* is essential for follicular development, and it also plays a key role in regulating ovulation in rats (McNatty et al., 2005).

Niu et al. (2021) worked on the importance of *BMP15* mutations affecting fertility in Cele black sheep in Xinjiang, China. The result showed that mutations are very useful and play an important role in breeding purposes in sheep.

Results of a study on Luzhong mutton sheep stated the association between litter size and *BMP15* as a major gene (Di et al., 2021).

Jiao et al. (2007) and Chu et al. (2007) reported that novel SNPs (A963G) and (C1050G), which were identified in exon 2 of *BMP15* and lead to amino acid changes in S300G and L329V, were associated with some fertility characteristics. In another study by Dong and Du (2010), the A901G SNP was investigated, which is in the same location as the A963G SNP; however they have used a different name to refer to it.

Interestingly, the mutation of the A963G of the *BMP15* gene (exon 2) in the Jining Grey, Lubei White and Yimeng Black goats was discovered to have a significant association with litter size.

**Table 6:**
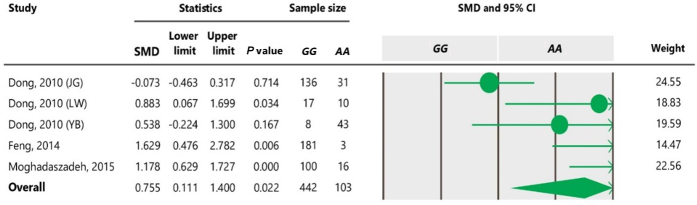
Forest plot for association between c.963A > G polymorphism and litter size under additive model. For additional details refer to Table 4 caption.

**Table 7:**
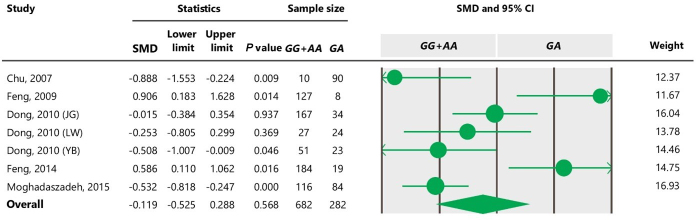
Forest plot of association between c.963A > G polymorphism and litter size under the co-dominant model. For additional details refer to the Table 4 caption.

**Table 8:**
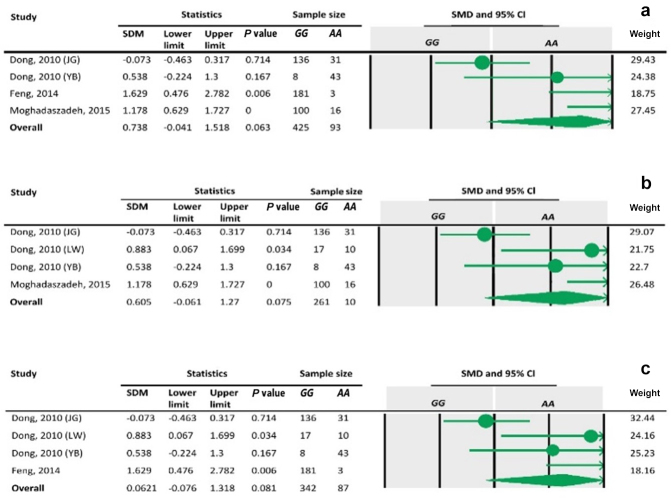
Forest plots of sensitivity analysis under the additive model, with the Dong and Du (2010) Lubei White breed study removed **(a)**, the Feng et al. (2014) study removed **(b)**, and the Moghadaszadeh et al. (2015) study removed **(c)**.

To the best of our knowledge, no meta-analysis has been conducted on the association of the A963G variant with litter size in goats. The meta-analyses of data under recessive and co-dominant models did not show evidence of association between the SNP and litter size (Tables 5 and 7). However, we observed significant association of A963G polymorphism with litter size under the dominant and additive models (Tables 4 and 6). In Table 4, the diamond lies entirely to the left side of the line of no effect, suggesting a significant difference in litter size between animals with *GG* and *GA* combined genotypes and those with the *AA* genotype ( P<0.05 ).

Nevertheless, the *GG* genotype differs from the *AA* genotype under an additive model (Table 6). For all genetic models, a random-effects model was used to analyze data because the obtained $I^{2}$ was more than 50 %, confirming existence of heterogeneity among studies. Our results showed that the allele A positively affects litter size in goats under dominant and additive genetic models. In the case of a co-dominant genetic model, the result indicated non-significant effects of genotypes on litter size using a random-effects model, but it was significant when a fixed-effects model was applied. It can be due to this fact that the inverse of the sum of within- and between-study variances is used in random-effects models, while only within-studies variances are used in fixed-effects models (Vesterinen et al., 2014). Consequently, we could not capture a significant difference between *GG* + *GA* combined genotypes and the *AA* genotype under a dominant genetic model by fitting the random-effects model. Higgins and Thompson (2002) suggested that random-effects models affect the confidence interval and the estimation of effective size. Further, heterogeneity probably results in a different pooled estimation, a wider confidence interval and a larger P value.

Finally, we performed a meta-analysis fitting a fixed-effects model to verify the results assessed using the random-effects model. The results showed a significant difference between *GG* + *GA* and *AA* genotypes under a dominant model ( P<0.05 ). The contrast we discovered between fixed-effects and random-effects models could be due to more equal weighting by random-effects models of studies included in the meta-analysis. The different weighting of studies by random-effects models in comparison with fixed-effects models causes the greater relative impact of small studies on the overall results of the meta-analysis.

To define the source of heterogeneity, we performed a sensitivity analysis by removing studies one by one. The results indicated that studies performed by Dong and Du (2010) on the Lubei White breed and by Feng et al. (2014) and Moghadaszadeh et al. (2015) influenced overall results of the meta-analysis by increasing the P value under an additive genetic model. One possible reason for this could be that studies with a larger Z value have SMD farther from overall SMD, which can lead to the increased P value.

In conclusion, the meta-analysis we have conducted has some advantages: (1) the data used in this meta-analysis were collected from all studies published in several languages; (2) for our meta-analysis study we have used four different genetic models to investigate association between c.963A > G polymorphism and litter size in goats, including dominant, recessive, additive and co-dominant models; and (3) through sensitivity analysis we have removed a single study at a time to validate the overall results. On the other hand, this meta-analysis had several limitations: (1) the limitation of the number of studies, which could affect the validity of overall results; (2) the sample sizes of studies we have used in this meta-analysis were small, and this may decrease the precision of obtained results; (3) we observed a high heterogeneity among studies under all four genetic models; and (4) the litter size could be affected by different factors such as other SNPs and genes, while we only investigated the effect of a single SNP (c.963A > G) on litter size in this meta-analysis.

## 5 Conclusions

Ultimately, the findings of the present meta-analysis study showed significant association between c.963A > G polymorphism and litter size in goats under dominant and additive genetic models. This meta-analysis suggested that genotype *AA* increases litter size in goats, but considering the limitations aforementioned, it is necessary to be careful in explaining the results of this meta-analysis.

## Data Availability

The original data from the paper are available from the corresponding author upon reasonable request.
